# Laparoscopy combining with ureteroscopy for horseshoe kidney accompanying with duplicate kidney and a ureteral calculus: a case report

**DOI:** 10.1186/s12894-020-00667-6

**Published:** 2020-07-11

**Authors:** Jindong Zhang, Liang Gao, Changlong Li, Xiaokang Yang, Yusheng Lei, Chuan Liu

**Affiliations:** Department of Urology, the Second Affiliated Hospital of Chongqing Medical University, Chongqing, 400000 China

**Keywords:** Horseshoe kidney, Duplicate kidney, Laparoscopy

## Abstract

**Background:**

Horseshoe kidney (HSK) is a common renal fusion anomaly, occurring in about 1 in 400–600 individuals. In addition, the incidence of duplicated collecting system is about 0.8%.

**Case presentation:**

This report documents an extremely rare case, which was treated by multiple procedures in the same operative session to accomplish laparoscopic amputation of the HSK isthmus, resection of duplicate kidney and ureteroscopic lithotripsy.

**Conclusion:**

Results showed that minimally invasive surgery with use of multiple endoscopes may be a feasible choice for this patient population with complicated comorbid renal conditions.

## Background

Horseshoe kidney (HSK) is a common renal fusion anomaly that typically involves the lower poles of the kidney and rarely the upper poles. This anomaly occurs in about 1 in 400–600 individuals, with a 2:1 male:female ratio [1, 2]. In addition, the occurrence of duplicated collecting system is about 0.8% and the upper poles of kidney are more frequently affected as a result of hydronephrosis [3]. However, the coexistence of these two anomalies is extremely rare [4]. This case report presents a patient who was diagnosed with HSK accompanied by duplicate kidney and ureteral calculus, which were treated by laparoscopy and ureteroscopy.

## Case presentation

In January 2019, a 31-year-old man was referred for evaluation at our hospital after the diagnosis of a giant renal cyst was made at another hospital, where the patient had initially presented with a 2-week history of dull right lower abdominal pain.

**Fig. 1:**
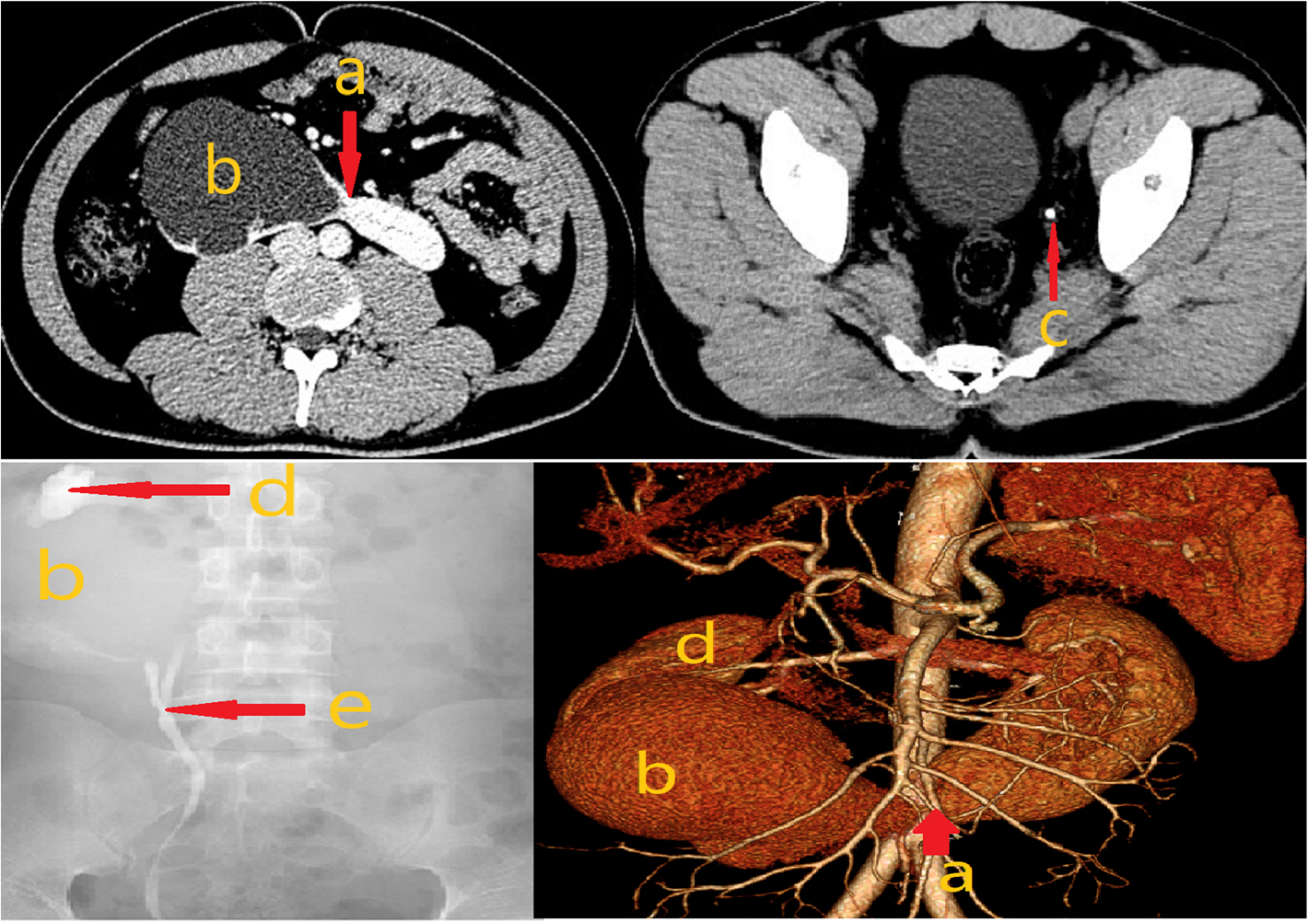
Abdominal enhanced computed tomography (CT), CT ureterography and retrograde urography images show: a) fusion of the bilateral lower poles; b) right lower kidney; c) left ureteral calculus; d) right upper renal collecting system; e) confluence of the duplicate renal collecting system

On admission, abdominal enhanced computed tomography (CT) combined with CT ureterography (CTU) showed the following: bilateral lower pole fusion; a low-density region (83 **×** 85 **×** 114 mm) in the right kidney during the arterial phase without entrance of the contrast agent in excretory phase and a left ureteral calculus with a 2.9-mm diameter. The multiple diagnoses of HSK with a right renal cyst and left ureteral calculus were suspected. (Fig. 1).

Considering the abnormal shape of renal cyst, however, we thought that the possibility of right hydronephrosis could not be eliminated. Therefore, a retrograde urography study was conducted, which showed a duplicated renal pelvis and ureters with severe hydronephrosis in the right lower renal pelvis (Fig. 1). Furthermore, results of renal imaging showed that the right lower kidney had no blood supply, whereas the right upper kidney had a normal blood supply; the glomerular filtration rates (GFRs) were calculated to be 0 mL/min and 23.7 mL/min, respectively. No other abnormalities were evident on other examinations. Based on these results, the diagnosis was confirmed as HSK accompanying a right duplicated kidney, severe hydronephrosis with a nonfunctioning right lower kidney and a left ureteral calculus.

### Surgery

**Fig. 2:**
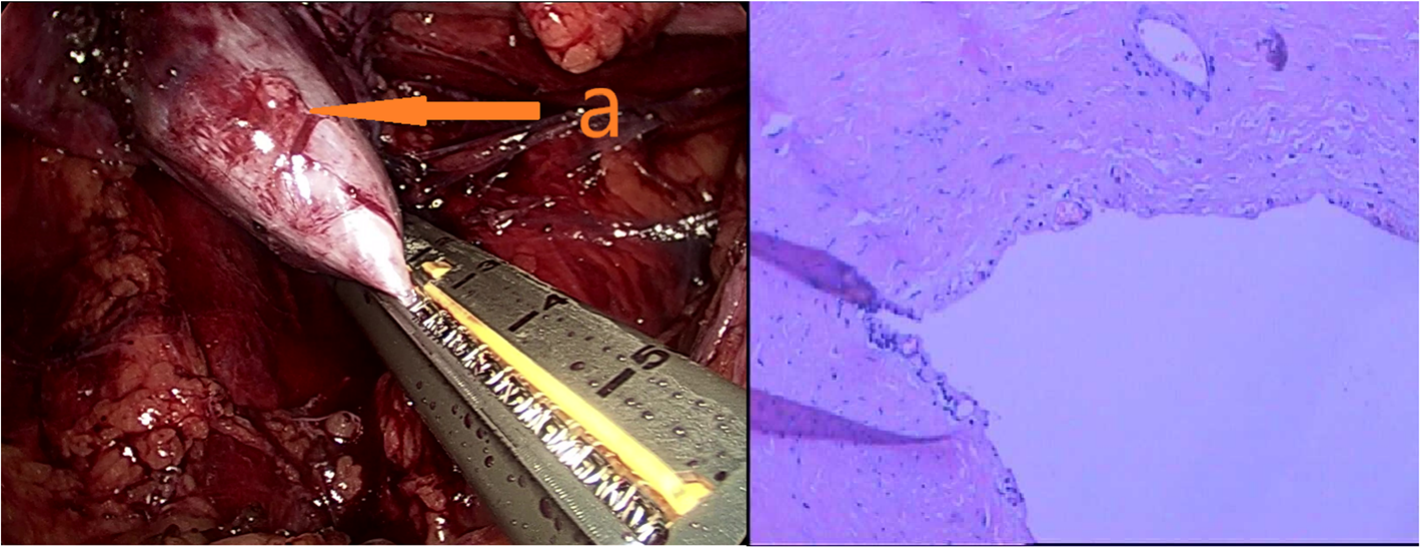
Laparoscopic dismemberment of the isthmus of the horseshoe kidney shows the right lower kidney (a) with hydronephrosis. Pathologic examination shows renal cortex atrophy, interstitial inflammatory cell infiltration, pyelomegaly and mucosal atrophy

Surgery was scheduled for the patient under general anesthesia. Firstly, the patient was placed in a bladder lithotomy position. A bilateral ureteroscope was used to remove the left ureteral calculus and stents were inserted into the ureters on each side. Secondly, the patient was placed in a modified lateral position and four puncture sheaths were inserted. After careful separation, a laparoscopic linear incision stapler was used to break off the isthmus of HSK (Fig. 2). Then, a knot-free suture (Johnson & Johnson) was used to reinforce the left side of separated section. After the inferior vessels were clamped and severed, the right lower kidney and ureter were excised.

During this procedure, however, the right ureteral stent was found to be inserted into the nonfunctional kidney. Therefore, this stent was removed through laparoscopy and then finally implanted through ureteroscopy in the bladder lithotomy position after the laparoscopic procedures.

### Outcomes

The operative time was 6 h and 12 min, Pathologic examination showed severe atrophy of the renal cortex and mucosa with the infiltration of inflammatory cells in interstitium (Fig. 2).

No obvious complications were observed except for moderate incision pain, which was relieved by an intravenous analgesic. The patient’s serum creatinine concentration returned to normal at postoperative Day 5, decreasing from the highest concentration on postoperative Day 2 at 125.5 umol/L. Abdominal X-ray showed that the position of the bilateral ureteral stents was normal. The patient was discharged on postoperative Day 6 after 13 hospitalisation days total.

**Fig. 3:**
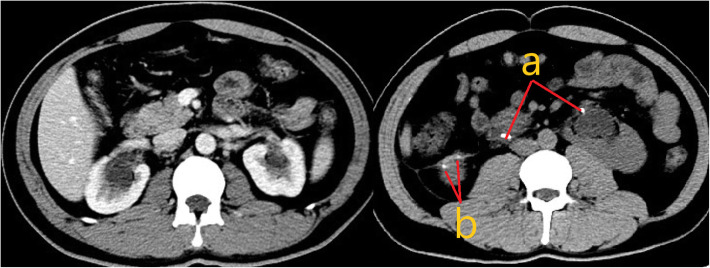
Postoperative computed tomography shows a few inflammatory changes and hydronephrosis for both kidneys: a) bilateral ureteral stent. b) postoperative changes after use of a laparoscopic linear incision stapler

At 3-month follow-up, the patient’s renal function remained normal. Abdominal CT showed a few inflammatory changes but no hydronephrosis for either kidney. The bilateral ureteral stents were successfully removed (Fig. 3).

## Discussion and conclusions

This case report presents the extremely rare anomaly of HSK comorbid with duplicated kidney. Most patients with duplicated kidney are asymptomatic and have no need for treatment [3]. However, these patients should be treated if complications occur, including hydronephrosis, calculus, infection or tumour [5]. In addition, most patients with HSK are also asymptomatic. Urinary tract infections, renal calculi and hydronephrosis are the most frequent complications, and 15–30% of patients have uretero-pelvic junction obstruction (UPJO) [6, 7]. The patient in this case had both anomalies, further complicated by hydronephrosis and a ureteral calculus, which was consistent with the complications noted in the literature.

A multicenter study reported that robot-assisted laparoscopic pyeloplasty (RALP) was a safe and feasible approach with satisfactory efficacy in children who had HSK [8]. A study by Guliev reported on 130 patients with HSK, 10 (7.7%) of whom had hydronephrosis treated with laparoscopic pyeloplasty, which achieved a success rate of 90% [9]. Minimally invasive surgery has gradually replaced traditional open surgery to shorten the hospital stay and postoperative recovery time; however. Retroperitoneal laparoscopic treatment for HSK is still rarely reported [10].

The standard treatment for duplicate kidney is surgical heminephrectomy [11]. Laparoscopic upper pole heminephrectomy was first reported in 1993, and then in several subsequent studies [12, 13]. Two different laparoscopic approaches have been used for the management of duplicate kidney, including the retroperitoneal approach in the lateral position and transperitoneal approach in the supine position [11]. Moreover, RALP is becoming a new minimally invasive method for treatment of duplicate kidney [14].

Up to now, only a few cases have been reported of HSK comorbid with duplicate kidney, but none of these patients have been treated laparoscopically. Because the experience in treating this patient population is very limited to date, the operative time and time that the stents were maintained was longer than that for more typical HSK or duplicate kidney cases. In addition, a ureteric stent was inserted to distinguish the duplicated ureter during surgery, which may serve to protect the normal ureter and avoid a possible stricture at the meeting of two ureters. Although this approach seemed to be working during laparoscopic procedure, the ureteric stent was subsequently found to have been inserted into the diseased ureter. and thus had to be reinserted after the laparoscopic procedure.

This case management was performed by laparoscopy in an improved position, which confirmed the effectiveness of this method. We hope this case presentation can provide a reference for the minimally invasive treatment of this disease. Based on the literature review and considering that to date only one such case has been treated by the approach described herein, the authors recommend that a highly experienced laparoscopic surgeon is required.

In conclusion, this report documents a rare case of multiple renal anomalies that included HSK, duplicate kidney, unilateral hydronephrosis and unilateral ureteral calculus. The results showed that minimally invasive surgery combining laparoscopy and ureteroscopy may be a safe and effective approach for this disease presentation.

## Data Availability

All data generated or analysed during this study are included in this published article.

## Abbreviations

HSK: horseshoe kidney
CT: computed tomography
CTU: computed tomography ureterography
GFR: glomerular filtration rate
UPJO: uretero-pelvic junction obstruction
PALP: robot-assisted laparoscopic pyeloplasty
